# Hypertonic saline reduces inflammation and enhances the resolution of oleic acid induced acute lung injury

**DOI:** 10.1186/1471-2466-8-9

**Published:** 2008-07-08

**Authors:** Muiris T Kennedy, Brendan D Higgins, Joseph F Costello, William A Curtin, John G Laffey

**Affiliations:** 1 Lung Biology Group, National Centre for Biomedical Engineering Sciences, National University of Ireland, Galway, Ireland; 2 Department of Surgery, Clinical Sciences Institute, National University of Ireland, Galway, Ireland; 3 Department of Anaesthesia, Clinical Sciences Institute, Galway University Hospitals and National University of Ireland, Galway, Ireland

## Abstract

**Background:**

Hypertonic saline (HTS) reduces the severity of lung injury in ischemia-reperfusion, endotoxin-induced and ventilation-induced lung injury. However, the potential for HTS to modulate the resolution of lung injury is not known. We investigated the potential for hypertonic saline to modulate the evolution and resolution of oleic acid induced lung injury.

**Methods:**

Adult male Sprague Dawley rats were used in all experiments. ***Series 1 ***examined the potential for HTS to reduce the severity of evolving oleic acid (OA) induced acute lung injury. Following intravenous OA administration, animals were randomized to receive isotonic (Control, n = 12) or hypertonic saline (HTS, n = 12), and the extent of lung injury assessed after 6 hours. ***Series 2 ***examined the potential for HTS to enhance the resolution of oleic acid (OA) induced acute lung injury. Following intravenous OA administration, animals were randomized to receive isotonic (Control, n = 6) or hypertonic saline (HTS, n = 6), and the extent of lung injury assessed after 6 hours.

**Results:**

In ***Series I***, HTS significantly reduced bronchoalveolar lavage (BAL) neutrophil count compared to Control [61.5 ± 9.08 versus 102.6 ± 11.89 × 10^3^ cells.ml^-1^]. However, there were no between group differences with regard to: A-a O2 gradient [11.9 ± 0.5 vs. 12.0 ± 0.5 KPa]; arterial PO2; static lung compliance, or histologic injury. In contrast, in ***Series 2***, hypertonic saline significantly reduced histologic injury and reduced BAL neutrophil count [24.5 ± 5.9 versus 46.8 ± 4.4 × 10^3^ cells.ml^-1^], and interleukin-6 levels [681.9 ± 190.4 versus 1365.7 ± 246.8 pg.ml^-1^].

**Conclusion:**

These findings demonstrate, for the first time, the potential for HTS to reduce pulmonary inflammation and enhance the resolution of oleic acid induced lung injury.

## Background

Hypertonic saline (7.5% saline, HTS) exhibits considerable potential as a therapeutic agent in diverse injury models. HTS has been demonstrated to inhibit acute lung injury (ALI) induced by systemic ischemia-reperfusion [1], hemorrhagic shock [2], and acute pancreatitis [3,4] in experimental models. The mechanisms of action of hypertonic saline are increasingly well understood, and include inhibition of neutrophil adhesion molecule CD11b [5], reduced TNF-α and IL- l production [1], and reduced activation of MAP kinase p38 and ERK-1 [6]. Pulmonary neutrophil sequestration, which is central to the mechanism of injury in ALI, is attenuated by HTS in diverse ALI models [1-3,7]. The therapeutic potential of HTS is demonstrated by its efficacy when used following initiation of the injury process, in both ischemia-reperfusion and pancreatitis induced ALI [1,3]. HTS has also demonstrated efficacy in clinical human trials, in patients with post-traumatic cerebral oedema [7].

Oleic acid induced lung injury is a well characterized and clinically relevant model of ALI/ARDS [8-12]. In particular, it constitutes an excellent model of Fat Embolism Syndrome (FES) induced ALI [13], given that OA is a major component of the marrow-derived fat emboli released into the circulation following traumatic bone injury [14,15]. Oleic acid produced a rapidly evolving lung injury [10], which features increased capillary permeability induced pulmonary edema [10] and alveolar infiltration of inflammatory cells [16]. The ALI produced by OA is relatively transient, and resolves over several hours [10]. This model therefore permits an evaluation of the therapeutic potential of HTS during both the injury and resolution phases of ALI [10].

The potential for HTS to modulate the resolution of lung injury is not known. We investigated the potential for hypertonic saline to modulate the evolution and resolution of oleic acid induced lung injury. Specifically, we hypothesized that HTS would modulate the severity of OA induced ALI, reducing lung injury and/or enhancing resolution of ALI. We used this model to determine the therapeutic potential of hypertonic saline in both evolving (Series 1) and resolving (Series 2) ALI.

## Methods

All experimental work was reviewed and approved by the Research Ethics Committee at the National University of Ireland, Galway and conducted under license from the Department of Health and Children, Dublin, Ireland.

### Animal Care

Specific pathogen free adult male Sprague Dawley rats (B&K Universal Ltd., U.K.) weighing 300 to 380 g were used in all experiments. Animals were housed for a minimum of 7 days prior to study, under controlled light/dark conditions where the light period was from 8:00 a.m. to 8:00 p.m. They were allowed access to ordinary rat chow and tap water *ad libitum*. Animals were maintained at constant ambient temperature of 21–22°C, while the humidity level was maintained between 45% and 55%.

### Fat Embolism Syndrome Model

The experimental model was based on that previously reported, with several modifications [8-12]. Briefly, anesthesia was induced and maintained with Isoflurane (Abbott Laboratories, UK). After confirming depth of anesthesia by absence of response to paw compression, a tail vein was cannulated using a 24 gauge cannula (Becton Dickinson, NJ). Oleic acid (OA) (Sigma-Aldrich, United Kingdom), which was prepared as a 1:1 mixture with pure ethanol [17], was administered intravenously. Animals were then randomized to hypertonic saline (HTS) or control groups. The HTS group were then administered 4 ml.Kg^-1 ^of 7.5% hypertonic saline and the control animals administered an equal volume of 0.9% saline. The animals were then allowed to recover from anesthesia, and then placed in their cages.

### Preliminary Studies

Two preliminary series of experiments was carried out to determine the dose of OA and duration of time required to produce a significant evolving ALI, and to produce a model of resolving lung injury. A dose of 40 μl of OA was determined to produce a significant early lung injury over a 4 h period. In later studies, a dose of 9 μl per 100 g body weight was found to produce a lung injury of similar magnitude at 4 hours, and which was resolving at 6 hours following OA administration.

### Series I – Evolving ALI

This series was carried out to determine the potential for HTS to attenuate evolving OA-induced injury. Following induction of anesthesia, 40 μl of OA was administered and each rat allowed to recover. Four hours following OA administration, the animals were re-anesthetized and the degree of ALI quantified.

### Series II – Resolving ALI

This series was carried out to determine whether HTS could modulate the resolution of OA-induced injury. Following induction of anesthesia, 9 μl.100 g^-1 ^body weight of OA was administered and each rat was allowed to recover. Six hours following OA administration, the animals were re-anesthetized and the extent of lung injury quantified.

### Assessment of Lung Injury

At four (Series I) or six (Series II) hours following OA administration, the animals were re-anesthetized with Isoflurane. After confirming depth of anesthesia by absence of response to paw compression, the dorsal penile vein and carotid artery were cannulated using 22G cannulae (Becton Dickinson, NJ). A tracheotomy was performed, and a tracheal tube was inserted to a depth of 2 cm and secured in place. Pancuronium (1 mg; Organon, The Netherlands) was administered intravenously and the lungs were then ventilated using a small animal ventilator (Model 683; Harvard Apparatus, United Kingdom) with a fractional inspired O_2 _(FiO_2_) of 0.21 (room air), respiratory rate 90 min.^-1^, tidal volume 4.5 ml.kg^-1 ^and 2 cm H_2_O positive end-expiratory pressure (PEEP).

Systemic arterial pressure, peak airway pressures and temperature were measured throughout the protocol. Arterial blood samples then were drawn for assessment of systemic oxygenation, ventilation and acid-base status (ABL 710, Radiometer, United Kingdom). Static inflation lung compliance was measured immediately prior to a recruitment manoeuvre, ensuring a standardized lung volume history. Incremental 1 ml volumes of room air were injected via the tracheal cannula, and the pressure attained 3 seconds after each injection was measured, until a total volume of 5 ml was injected. At the end of the protocol, the inspired gas was altered to FiO_2 _of 1.0 for 5 minutes, and an arterial blood sample was then taken for calculation of alveolar-arterial oxygen gradient. The animals were then euthanized by exsanguination under anesthesia.

### Sampling and Assay Protocol

Immediately post mortem, the heart-lung block was dissected from the thorax and bronchoalveolar lavage (BAL) performed. BAL was carried out by intratracheal instillation of 3 aliquots (5 ml each) of isotonic saline and collection of the returned fluid by free drainage. Total cell numbers per ml in BAL fluid were counted and differential cell counts were performed following staining with Diff-Quik (BDH, United Kingdom). Samples of BAL fluid were centrifuged, snap frozen in liquid N_2 _and stored at -80°C. The concentration of interleukin-6 (IL-6) in these BAL samples was determined using quantitative sandwich ELISA's (R&D Systems, United Kingdom). The BAL protein concentration was determined using a standard assay (Biorad Assay, Biorad, Hercules, CA).

The left lung was isolated and fixed with 4% paraformaldehyde [18,19], and the extent of histologic lung damage determined using quantitative stereological techniques as previously described [20].

### Statistical Analysis

Results are expressed as mean ± (SEM) for normally distributed data, and as median (interquartile range, IQR) if non-normally distributed. Data were analyzed by one-way ANOVA followed by Student-Newman-Keuls, t-test or Kruskalis-Wallis followed by Mann-Whitney U test with the Bonferroni correction for multiple comparisons, as appropriate. P < 0.05 was considered statistically significant.

## Results

### Series 1 – Evolving OA induced ALI

12 animals were entered into this study. All animals entered into the study survived the full duration of the protocol.

#### Physiologic Indices of Lung Damage

There was no difference between the groups with regard to arterial oxygen tension, or alveolar-arterial gradient at 21% or 100% oxygen [Table 1]. There was no difference in peak airway pressures or in static lung compliance between the groups [Table 1].

**Table 1:** Data for evolving ALI series

**Variable**	**Hypertonic Saline (n = 6)**	**Control (n = 6)**
Animal Weight (g)	354 ± 10.0	336 ± 5.3
Mean Arterial Pressure (mmHg)		
0 min	144 ± 12.0	140 ± 7.4
10 min	167 ± 7.4	149 ± 7.6
20 min	158 ± 8.7	148 ± 7.8
Peak Airway Pressure (mmHg)		
0 min	5.4 ± 0.6	5.7 ± 0.2
10 min	5.3 ± 0.5	5.7 ± 0.2
20 min	6.1 ± 0.6	6.5 ± 0.2
Arterial PO_2_		
FiO_2 _0.3	11.9 ± 0.5	12.0 ± 0.5
FiO_2 _1.0	72.0 ± 1.5	71.7 ± 2.1
Alveolar-arterial O_2 _Gradient (mmHg)		
FiO_2 _= 0.21	11.9 ± 1.00	10.9 ± 0.56
FiO_2 _= 1.0	21.8 ± 4.94	17.3 ± 2.25
Static Lung Compliance (ml.mmHg^-1^)	0.43 ± 0.02	0.44 ± 0.02
BAL Protein concentration (mg/L)	4559 ± 418	5398 ± 522

**Notes: **Data are expressed as mean ± SEM.

*Significantly different from Hypertonic Saline (P < 0.02, ANOVA).

#### Lung Damage and Inflammation

**Figure 1:**
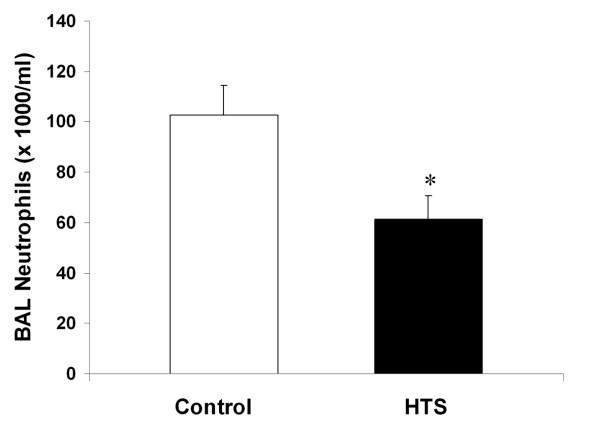
**Histogram representing number of neutrophils per mililitre of bronchoalveolar lavage fluid in evolving oleic acid induced ALI**. The abbreviations used are as follows: HTS, hypertonic saline; BAL, bronchoalveolar lavage. * Significantly different compared to control (P < 0.05, t test).

**Figure 2:**
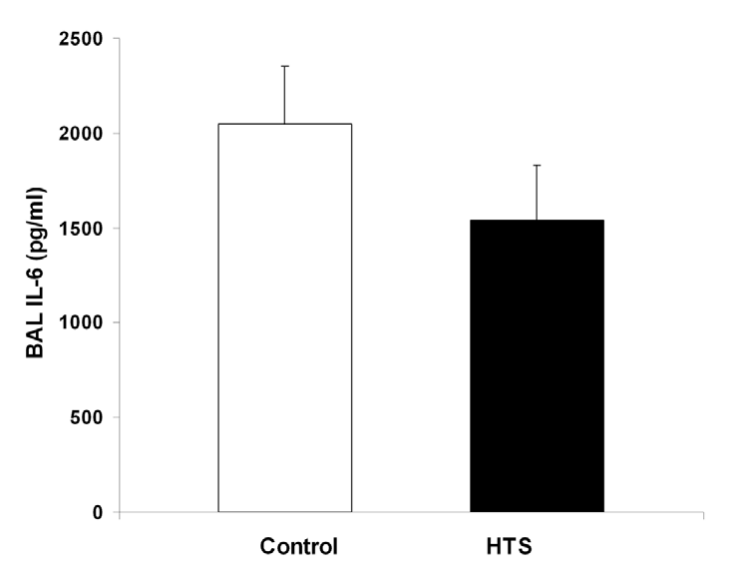
**Histogram representing the bronchoalveolar lavage interleukin 6 concentrations in evolving oleic acid induced ALI**. The abbreviations used are as follows: HTS, hypertonic saline; BAL, bronchoalveolar lavage.

**Figure 3:**
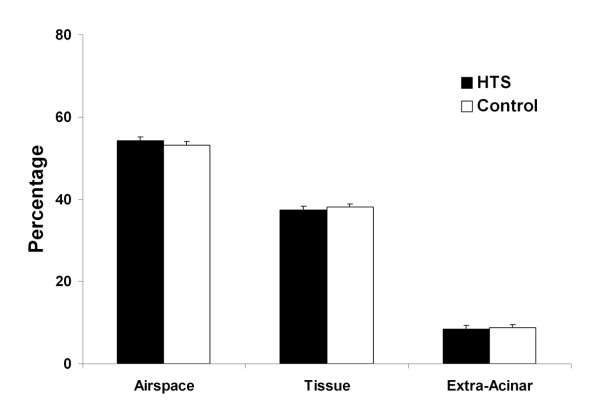
**Histogram representing stereologic assessment of the extent of histologic injury in evolving oleic acid induced ALI**. The abbreviations used are as follows: HTS, hypertonic saline.

HTS significantly reduced BAL neutrophil counts compared to control conditions [Figure 1]. BAL IL-6 [Figure 2], and BAL protein levels [Table 1] were reduced with HTS, although these differences were not statistically significant [Figure 3]. However, there was no between group difference in the degree of histologic injury. Quantitative stereological analysis demonstrated that there was no significant difference between HTS and Control in regard to the amount of tissue or airspace in the gas exchanging portion of the lung.

### Series 2 – Resolving OA induced ALI

24 animals were entered into this study. All animals entered into the study survived the full duration of the protocol.

#### Physiologic indices of Lung Damage

There was no difference between the groups with regard to arterial oxygen tension, or alveolar-arterial gradient at 21% or 100% oxygen [Table 2]. There was no difference in peak airway pressures or in static lung compliance between the groups [Table 2].

**Table 2:** Data for resolving ALI series

**Variable**	**Hypertonic Saline (n = 12)**	**Control (n = 12)**
Animal Weight (g)	330 ± 6.5	334 ± 5.5
Mean Arterial Pressure (mmHg)		
0 min	141 ± 9.0	119 ± 8.9
10 min	160 ± 7.3	150 ± 5.8
20 min	150 ± 7.8	142 ± 7.5
Peak Airway Pressure (mmHg)		
0 min	4.8 ± 0.3	5.2 ± 0.2
10 min	4.7 ± 0.3	4.9 ± 0.1
20 min	5.1 ± 0.3	5.3 ± 0.2
Arterial PO_2_		
FiO_2 _0.3	12.2 ± 0.6	11.9 ± 0.6
FiO_2 _1.0	71.0 ± 0.9	68.3 ± 2.0
Alveolar-arterial O_2 _Gradient (mmHg)		
FiO_2 _= 0.21	10.8 ± 0.5	11.0 ± 0.6
FiO_2 _= 1.0	18.7 ± 0.9	22.0 ± 2.0
Static Lung Compliance (ml.mmHg^-1^)	0.55 ± 0.04	0.44 ± 0.04
BAL Protein concentration (mg/L)	2595 ± 640	3888 ± 564

Data are expressed as mean ± SEM.

*Significantly different from Hypertonic Saline (P < 0.05, ANOVA).

#### Lung Damage and Inflammation

**Figure 4:**
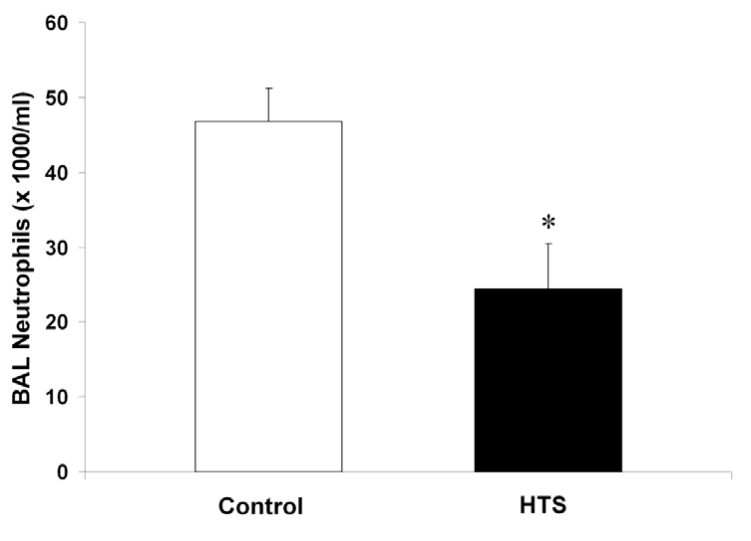
**Histogram representing number of neutrophils per mililitre of bronchoalveolar lavage fluid in resolving oleic acid induced ALI**. The abbreviations used are as follows: HTS, hypertonic saline; BAL, bronchoalveolar lavage. * Significantly different compared to control (P < 0.05, t test).

**Figure 5:**
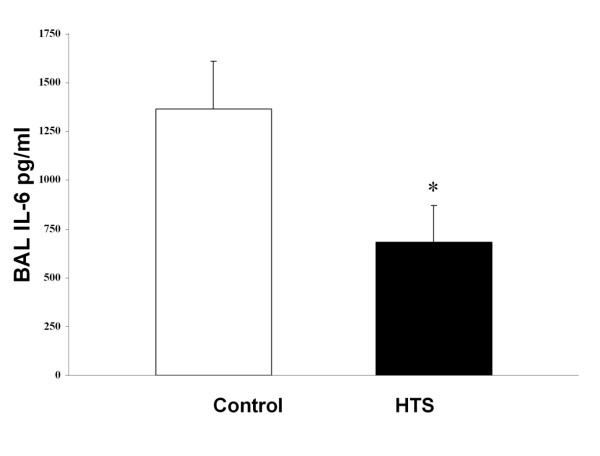
**Histogram representing the bronchoalveolar lavage interleukin 6 concentrations in evolving oleic acid induced ALI**. The abbreviations used are as follows: HTS, hypertonic saline; BAL, bronchoalveolar lavage. * Significantly different compared to control (P < 0.05, t test).

**Figure 6:**
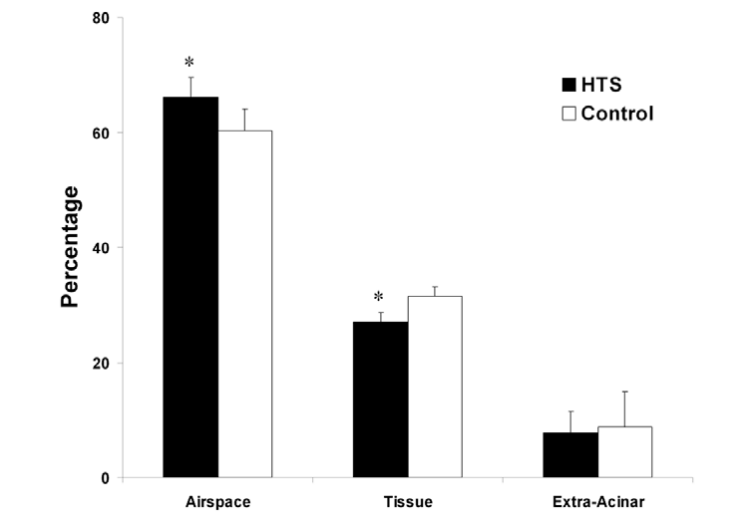
**Histogram representing stereologic assessment of the extent of histologic injury in evolving oleic acid induced ALI**. The abbreviations used are as follows: HTS, hypertonic saline. * Significantly different compared to control (P < 0.05, t test).

HTS significantly reduced BAL neutrophil counts [Figure 4], and BAL IL-6 concentrations [Figure 5] compared to control conditions. BAL protein levels were reduced with HTS, although these differences were not statistically significant [Table 2]. HTS significantly reduced the degree of histologic injury compared to isotonic saline [Figure 6]. Quantitative stereological analysis demonstrated that HTS significantly increased alveolar airspace, and significantly reduced alveolar tissue, indicating reduced histologic lung damage, compared to control conditions.

## Discussion

We demonstrate for the first time the potential for hypertonic saline to modulate the resolution of acute lung injury. Hypertonic saline reduced alveolar neutrophil infiltration in both evolving and resolving oleic induced ALI. Of perhaps greater significance, HTS enhanced the resolution of OA induced lung injury, as evidenced by a reduced severity of histologic injury, and reduced indices of inflammation, compared to control conditions.

### HTS – Therapeutic Potential in ALI

The neutrophil-endothelial cell interaction is central to the pathogenesis of ALI/ARDS. HTS is a potent inhibitor of neutrophil function, downregulating neutrophil oxidative burst activity [21], reducing neutrophil adhesion molecule expression [22] and suppressing neutrophil activation and the release of pro-inflammatory cytokines [5,23]. HTS decreased lung neutrophil infiltration [24,25] and ICAM-1 [25] expression in the setting of hemorrhagic shock.

HTS has been demonstrated to reduce pulmonary edema, histologic injury, and lung neutrophil infiltration following systemic ischemia-reperfusion injury [1]. HTS also reduced serum IL-6 and TNF-α cytokine levels in this study. HTS reduced the increase in lung permeability and lung neutrophil sequestration and activation following trauma-haemorrhagic shock [26]. HTS resuscitation also increased survival, and reduced lung injury, in a two-hit injury model of hemorrhage followed by cecal ligation and puncture in mice [27]. HTS transiently improved tissue oxygen delivery and increased oxygen consumption in a canine model of oleic acid-induced lung injury [28]. However, the potential for HTS to modulate oleic acid-induced ALI has not been assessed

### Effect of HTS on evolving ALI

HTS did not reduce the severity of evolving oleic acid induced ALI. There was no difference between HTS and an equal volume of isotonic saline in terms of arterial oxygenation, pulmonary shunt or lung static or dynamic compliance. The degree of histologic injury on quantitative stereological analysis was similar with HTS versus isotonic saline, with no difference in the amount of tissue or airspace in the gas exchanging portion of the lung. In contrast to these findings, HTS did appear to reduce pulmonary inflammation, significantly reducing bronchoalveolar lavage neutrophil cell counts, in keeping with previous findings in hemorrhage-induced ALI models [22,24,25]. While the bronchoalveolar lavage protein and interleukin-6 levels showed a clear trend towards decrease in the HTS group, the results were not statistically significant. Therefore, while HTS reduced indices of pulmonary inflammation, it not attenuate the severity of oleic acid induced ALI.

### HTS enhances resolution of ALI

A key feature of the oleic acid model of ALI is the fact that the injury is transient, permitting study of the effects of HTS on the resolution of the ALI. HTS did enhance the resolution of oleic acid induced ALI. Of importance, the degree of histologic injury on quantitative stereological analysis was clearly reduced by HTS. Specifically, there was a significantly reduced amount of tissue and a significantly greater amount of airspace in the gas exchanging portion of the lung. Furthermore, HTS reduced pulmonary inflammation, significantly reducing bronchoalveolar lavage neutrophil cell counts and interleukin-6 levels. Interestingly, HTS did not modulate physiologic indices of lung injury, in terms of arterial oxygenation, pulmonary shunt or lung static or dynamic compliance. This may reflect the fact that different parameters of lung injury and damage may resolve over differing time courses.

### Limitations of findings

Our study has a number of limitations. In particular, it is not clear whether the effects seen are a function of the additional saline load, or the hypertonicity per se. We chose to use a control group of an equal volume of isotonic saline in order to compare the effects of equal infusion volumes. Additional studies, which compare the effects of HTS to an equi-osmolar load of isotonic saline, are required. The relatively short experimental time frames studied may have restricted the capacity to fully characterize the therapeutic potential of HTS, particularly during the recovery phase. However oleic acid injury is relatively transient and the time points utilized allowed characterisation of the effects of HTS during injury evolution and resolution.

## Conclusion

In conclusion, we report for the first time that hypertonic saline enhances the resolution of oleic induced ALI. These findings further enhance the therapeutic potential of hypertonic saline in ALI/ARDS.

## Abbreviations

A-a O_2 _gradient: Alveolar-arterial oxygen gradient; ALI: Acute Lung Injury; ANOVA: analysis of variance; ARDS: Acute Respiratory Distress Syndrome; BAL: Bronchoalveolar Lavage; °C: degrees centigrade; ERK-1: extracellular receptor kinase-1; FiO_2_: Fractional inspired oxygen concentration; g: grams; HTS: Hypertonic saline; ICAM: Intercellular adhesion molecule; IL: Interleukin; KPa: KiloPascal; MAP: Mitogen activated protein; μL: microlitres; ml.Kg^-1^: mililiters per kilogram; min: minutes; OA: Oleic acid; PO_2_: Partial pressure of Oxygen; SEM: Standard error of the mean; TNF-α: Tumor necrosis alpha.

## Competing interests

The authors declare that they have no competing interests.

## Authors' contributions

MTK conceived of the study, and participated in its design and execution and helped to draft the manuscript. BDH participated in the study execution and the histologic analysis, and helped to draft the manuscript. JFC participated in the histologic analysis and helped to draft the manuscript. JGL participated in the design and coordination of the study, performed the statistical analysis, and helped to draft the manuscript. All authors read and approved the final manuscript.

## Pre-publication history

The pre-publication history for this paper can be accessed here:


